# Machine-learned dimethyl sulphide (DMS) for the North Atlantic (2002–2024) to support movement studies

**DOI:** 10.1038/s41598-026-55205-5

**Published:** 2026-06-02

**Authors:** Meixuan Liu, Fernando Benitez-Paez, Oliver Padget, Dmitry Kishkinev, Urška Demšar

**Affiliations:** 1 School of Geography and Sustainable Development, University of St Andrews, St Andrews, UK; 2 Department of Earth, Ocean and Ecological Sciences, University of Liverpool, Liverpool, UK; 3 School of Life Sciences, Keele University, Newcastle, UK

**Keywords:** Dimethyl sulphide, Machine learning, Bird movement, Olfactory navigation, Ensemble method, Ecology, Ecology, Environmental sciences, Ocean sciences

## Abstract

Dimethyl sulphide (DMS) serves as a key olfactory cue for seabird navigation, yet existing DMS products operate at coarse spatiotemporal resolutions (≥ 25 km, monthly) mismatched to the scales of individual movement decisions. We ask (I) whether machine learning can produce biologically relevant, high-resolution DMS estimates across the North Atlantic, and (ii) whether such estimates can be readily integrated with animal tracking data to support ecological interpretation of animal trajectories. Using North Atlantic in-situ DMS observations (2002–2024) and five satellite-data-based environmental predictors (chlorophyll, mixed layer depth, nitrate, sea-surface temperature, and photosynthetically available radiation), we developed a machine-learning-based ensemble model for DMS prediction that achieved strong accuracy (test R^2^ = 0.88; RMSE = 0.859 µmol m^−3^), exceeding previously reported performance for basin-scale DMS mapping. To identify the key drivers of the model predictions, we conducted SHAP (SHapley Additive exPlanations) analysis, which revealed that mixed layer depth, nitrate concentration, and chlorophyll were the dominant controlling factors, aligning with established understanding of DMS biogeochemistry. We then produced a spatially continuous, daily 4 km DMS dataset for the North Atlantic domain (0–60° N, 80° W–15° E), revealing seasonal cycles, persistent hotspots, and fine-scale gradients not captured by coarse climatologies. Finally, we develop AniDMS, an open-source Python package that automates trajectory annotation with gridded DMS and associated covariates, demonstrated with a Manx shearwater case study. Together, the dataset and the tool enable scalable, hypothesis-driven tests of olfactory navigation of seabirds and provide a transferable framework for integrating high-resolution environmental context into movement ecology.

**Supplementary Information:**

The online version contains supplementary material available at 10.1038/s41598-026-55205-5.

## Introduction

Understanding seabird navigation requires environmental information that matches the spatial and temporal scales at which birds move and make decisions. Despite strong evidence that *Procellariiform* seabirds rely on olfactory cues, particularly dimethyl sulphide (DMS), to guide long-distance movements at sea, existing DMS datasets remain poorly suited for testing navigation hypotheses. Individual seabirds routinely travel tens to hundreds of kilometres per day, yet available DMS products are typically monthly and spatially coarse, often exceeding 25 km resolution. This mismatch has constrained empirical evaluation of olfactory navigation in the marine environment.

Navigation in birds is generally framed within a map and compass system^1–4^, in which individuals estimate their position relative to a goal using large scale environmental gradients and orient using directional cues^5–8^. Birds integrate multiple sensory modalities, including geomagnetic, visual, acoustic, and olfactory information, with cue use varying across species and ecological contexts^9–12^. While geomagnetic and celestial cues have received extensive attention, olfaction has emerged as a critical but comparatively less studied component of avian navigation in wild birds^13–17^, particularly for pelagic species where visual landmarks are scarce and environmental gradients may be more stable^14,18^.

*Procellariiform* seabirds represent a compelling model for large scale olfactory navigation. These species possess highly developed olfactory bulbs and associated neural structures, and experimental studies demonstrate that olfaction is essential for both foraging and homing to nesting burrows across vast oceanic regions^14,18–22^. Flight path analyses further suggest movement patterns consistent with map like navigation guided by chemical gradients. In contrast, geomagnetic cues appear neither sufficient nor necessary for navigation in many pelagic seabirds^23–26^, reinforcing the importance of alternative sensory mechanisms in the open ocean^15,27^.

Among olfactory cues, DMS has emerged as a key compound linking marine biogeochemistry and seabird behaviour. DMS is a biogenic sulphur compound released during phytoplankton grazing and is associated with regions of elevated primary and secondary productivity^28^. *Procellariiform* seabirds are attracted to DMS during foraging, and experimental evidence indicates that exposure to DMS influences orientation and movement decisions^22–25^. In the marine environment, DMS is particularly well suited as a navigational cue. Compared to terrestrial odours that are readily disrupted by turbulence and landscape heterogeneity, the open ocean provides a relatively stable medium for chemical dispersion, allowing regional scale gradients to persist over time^22,23,29–31^. These properties make DMS a plausible signal for both locating productive foraging areas and supporting large scale navigation^32^.

However, testing the navigational role of DMS in wild seabirds has been limited by the availability of suitable environmental data. Characterising DMS concentration fields requires understanding spatial distribution and spatiotemporal dynamics, resolved at scales relevant to animal movement^33^. In situ measurements of DMS are sparse and unevenly distributed, reflecting historical survey effort and methodological heterogeneity^34^. As a result, most existing DMS datasets have been developed for climatological or biogeochemical applications rather than movement ecology.

Current DMS estimation approaches fall into three broad categories. (i) Parameterisation-based and empirical remote sensing models infer DMS from relationships with environmental variables such as chlorophyll, mixed layer depth, and sea surface temperature, providing global or regional climatologies^35–41^. (ii) Interpolation-based products extrapolate discrete measurements to continuous surfaces through spatial smoothing^42,43^. (iii) More recently, machine learning approaches, including artificial neural networks^44,45^, random forest regression^46^, and Gaussian process regression^47^, have improved predictive accuracy relative to earlier methods. Despite these advances, existing models largely operate at monthly temporal resolution and coarse spatial scales (≥ 25 km), reflecting their primary use in estimating long term biogeochemical fluxes rather than dynamic environmental gradients encountered by moving animals.

This limitation is particularly relevant in the North Atlantic, which hosts major *Procellariiform* populations, including Manx shearwaters (*Puffinus puffinus*), which we used for showcasing our Python tool, yet remains underrepresented in high-resolution DMS modelling efforts^45^. The region exhibits strong seasonal dynamics and spatial heterogeneity, suggesting that coarse climatologies may smooth over continuous DMS gradients and obscure biologically meaningful variation. As individual birds move tens to hundreds of kilometres daily, they are likely to spot and respond to fine-scale DMS gradients along their flight paths and within foraging areas. To test hypotheses about olfactory guided navigation, DMS data therefore need to capture daily variability and resolve fine scale gradients along migratory and foraging routes.

Here, we address this gap by developing a machine learning based approach to estimate DMS at spatial and temporal scales aligned with seabird movement. We ask two related questions: Can high-resolution DMS prediction enable ecological interpretation of seabird trajectories? And how can machine learning derived DMS estimates be effectively integrated with tracking data to support navigation research? We develop an ensemble machine learning model that achieves improved predictive performance across the North Atlantic. Using this model we generate and openly publish the first daily DMS dataset at 4 km resolution covering the period 2002 to 2024. To facilitate ecological applications, we further introduce AniDMS, an open-source Python package that enables automated annotation of movement trajectories with our high-resolution DMS data. These contributions also enable previously unexplored tests of olfactory navigation hypotheses at spatial and temporal scales relevant to seabird movement.

## Results

### High-resolution DMS prediction model

#### Model performance

We evaluated nine machine learning models for DMS prediction, comprising six individual base learners and three stacking ensemble configurations with different meta-learners (Table 1). Among base learners, tree-based ensemble models performed best. Random Forest, XGBoost, and LightGBM achieved test R^2^ values of 0.875, 0.873, and 0.854, respectively, with test RMSE between 0.88 and 0.95 µmol m^−3^. In contrast, kernel-based models (SVR, GPR) and the neural network (MLP) showed substantially lower predictive skill, with test R^2^ below 0.75.

**Fig. 1:**
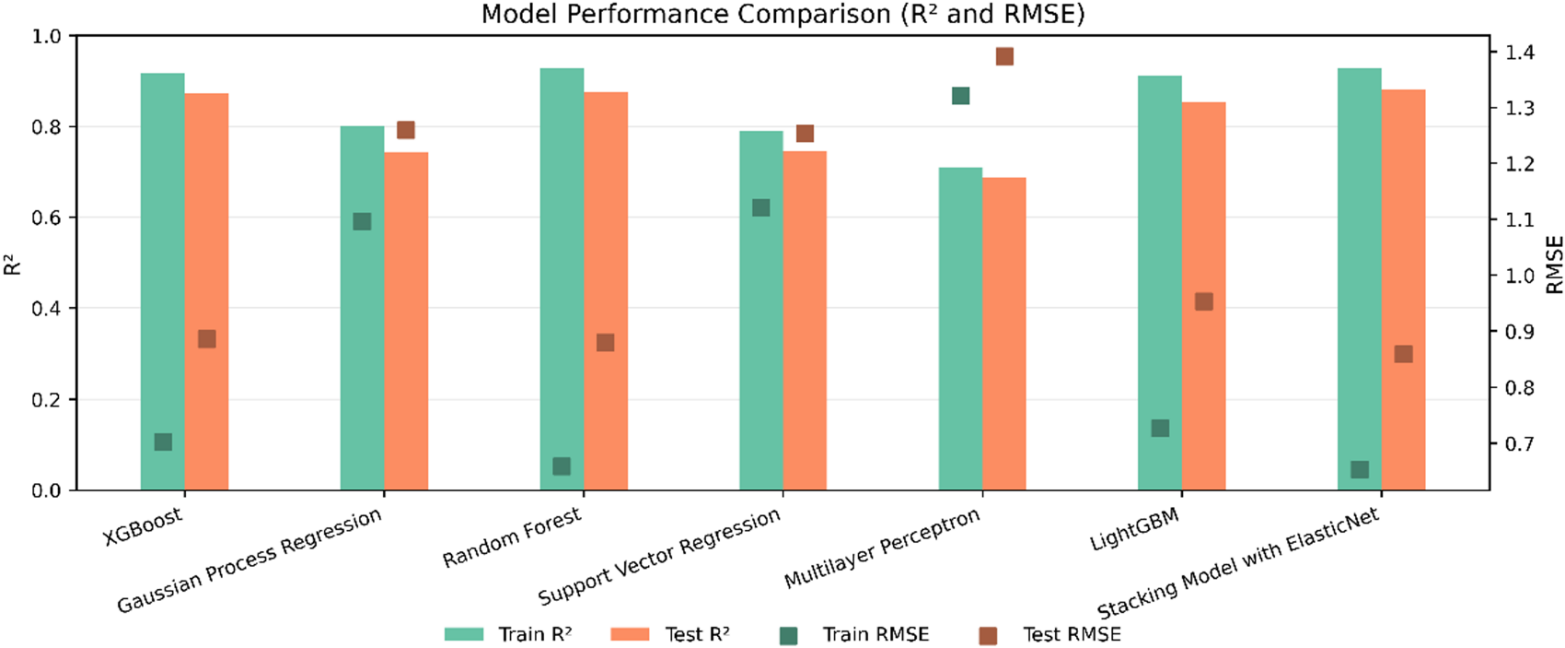
Model performance comparison across training and testing datasets. Bars represent R² values (primary y-axis) and squares represent RMSE values (secondary y-axis, µmol m^−3^) for each algorithm. Dark/light green indicates training performance, while orange/brown indicates testing performance.

All stacking ensembles improved test performance relative to individual base learners, although the choice of meta-learner strongly influenced generalisation. Ridge-based stacking exhibited clear overfitting, with high training performance but degraded test accuracy. Lasso-based stacking achieved strong test performance but retained a pronounced train–test discrepancy. The ElasticNet stacking model provided the most balanced performance, achieving the highest test accuracy (R² = 0.881, RMSE = 0.859 µmol m^−3^) while maintaining a small gap between training and testing metrics. This model was therefore selected for subsequent analyses and for generating the North Atlantic DMS dataset. Performance differences across models are summarised in Fig. 1 and Table 1.

**Table 1:** Model performance for machine learning algorithms.

Model	Train RMSE	Train *R*^2^	Test RMSE	Test *R*^2^
Random forest (RF)	0.6581	0.9281	0.8798	0.8751
XGBoost	0.7028	0.9180	0.8858	0.8734
Light GBM (LGBM)	0.7265	0.9124	0.9527	0.8535
Support vector regression (SVR)	1.1211	0.7914	1.2537	0.7464
Gaussian process regression (GPR)	1.0963	0.8005	1.2594	0.7440
Multi-layer perceptrons (MLP)	1.3215	0.7101	1.3917	0.6874
Stacking with ridge regression	0.8988	0.9375	1.1768	0.8687
Stacking with lasso regression	0.7003	0.9587	0.8730	0.8800
Stacking with ElasticNet	0.6529	0.9292	0.8592	0.8809

#### Feature importance and environmental drivers (SHAP)

**Fig. 2:**
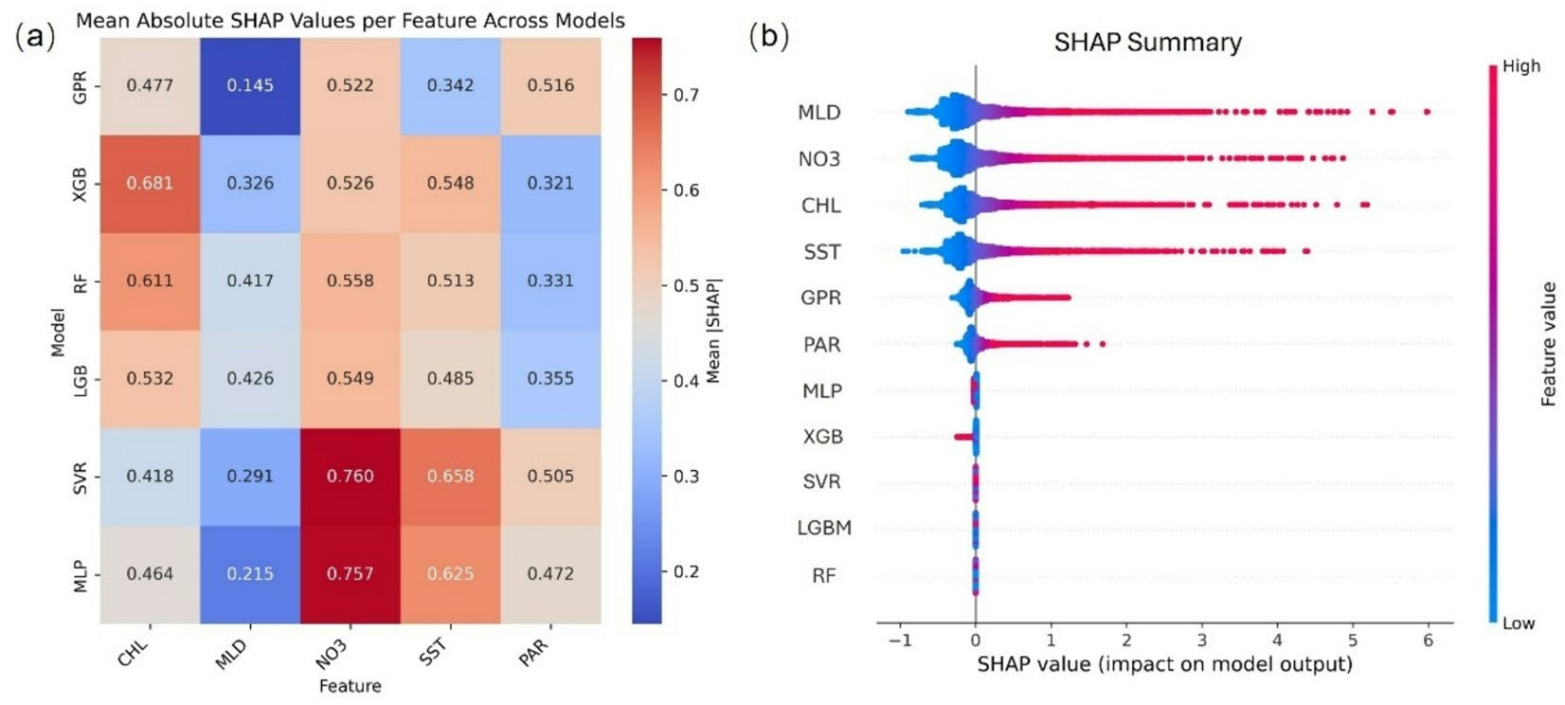
SHAP analysis reveals environmental drivers of DMS prediction. **a** Mean absolute SHAP values summarising feature importance across models. **b** SHAP beeswarm plot for the ElasticNet stacking model, showing nonlinear effects of key predictors. Colours indicate feature values, and positive SHAP values correspond to increased DMS predictions.

SHAP analysis was used to identify key environmental drivers of DMS variability across individual base learners and the ensemble model (Fig. 2). For base models, nitrate concentration (NO_3_), chlorophyll-a (CHL), and sea surface temperature (SST) consistently ranked among the most influential predictors, particularly for tree-based models (Fig. 2a). Kernel-based and neural network models showed greater sensitivity to photosynthetically available radiation, while Gaussian Process Regression emphasised NO₃ and CHL.

Feature importance rankings were broadly consistent within model groups, with tree-based models showing particularly high agreement (Spearman’s ρ > 0.85; Supplementary Fig. S1). Because the ElasticNet stacking ensemble achieved the strongest predictive performance among the evaluated models (Table 1), we focus interpretation on its SHAP patterns. In this model, mixed layer depth (MLD) emerged as the dominant predictor, followed by NO₃, CHL, and SST (Supplementary Fig. S2). The SHAP analysis (Fig. 2b) revealed complex nonlinear relationships between environmental features and DMS predictions. Deeper mixed layers were strongly associated with elevated DMS concentrations, consistent with mechanisms that a deeper mixed layer brings abundant nutrient entrainment and supports surface phytoplankton bloom with high productivity. High NO_3_, CHL and SST similarly drove positive predictions, reflecting nutrient-enhanced DMS production pathways. Also, these drivers align with the baseline GPR study used for comparison, where a multiple linear regression analysis identified CHL and MLD as key environmental correlates of DMS. Our results, therefore, reproduce similar dominant controls while extending them to daily, 4-km predictions and allowing for non-linear effects.

Predictions from a weaker base learner (GPR) contributed non-negligibly to the ensemble, indicating that stacking effectively integrates complementary information beyond the strongest individual models. Detailed SHAP results for individual base learners are provided in Supplementary Fig. S3-S8.

#### Robustness and generalisation

To assess robustness across the North Atlantic, the ElasticNet stacking model and all base learners were applied to predict DMS at all available observation locations (Fig. 4). Although the model was trained and evaluated using observations spanning 0–75°N, the final data product is restricted to 0–60°N due to limitations in satellite data availability during polar night conditions (Sect. 5.1).

Across all observations, the stacking model demonstrated strong generalisation with minimal systematic bias. Within 0–60°N, predictive performance reached RMSE = 0.721 µmol m^−3^ and R^2^ = 0.915, with only marginal degradation when extended to 0–75°N. Predictions clustered tightly around the 1:1 line across the full concentration range, including elevated DMS values associated with bloom conditions. Tree-based models showed greater dispersion, while Bayesian-based and neural network models exhibited significant scatter, particularly at high concentrations. Underestimation of extreme DMS values was observed across all models, but was least pronounced in the stacking ensemble. The minimal performance difference between the 0–60°N and 0–75°N ranges (∆RMSE = 0.014 µmol m^−3^) indicates that the model generalises well to higher latitudes despite sparser training data in those regions. Overall comparisons are provided in Supplementary Fig. S9.

**Fig. 3:**
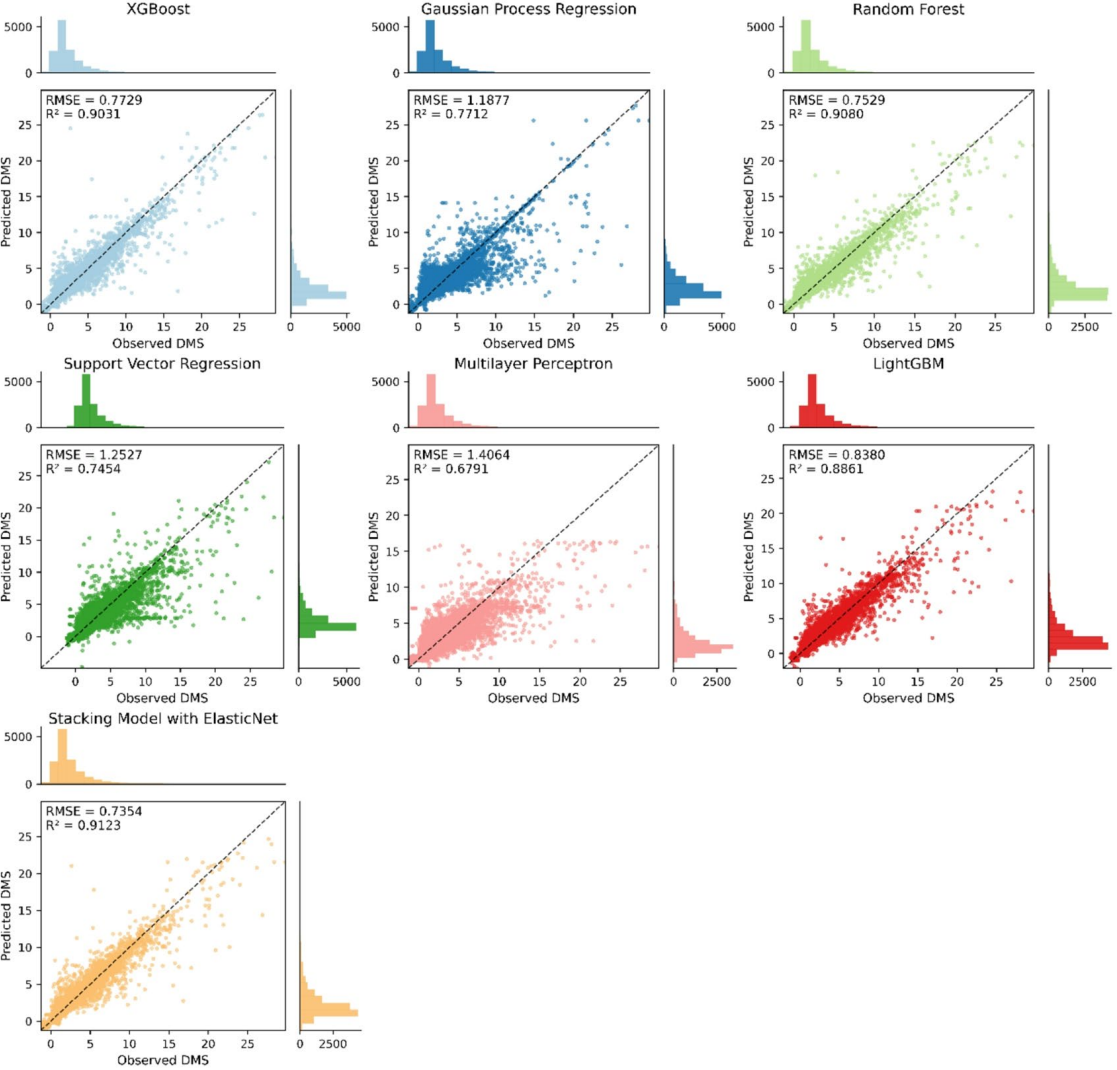
Observed versus predicted DMS concentrations across the North Atlantic. Each point represents a DMS measurement, with colours indicating predictions from different machine learning algorithms.

**Fig. 4:**
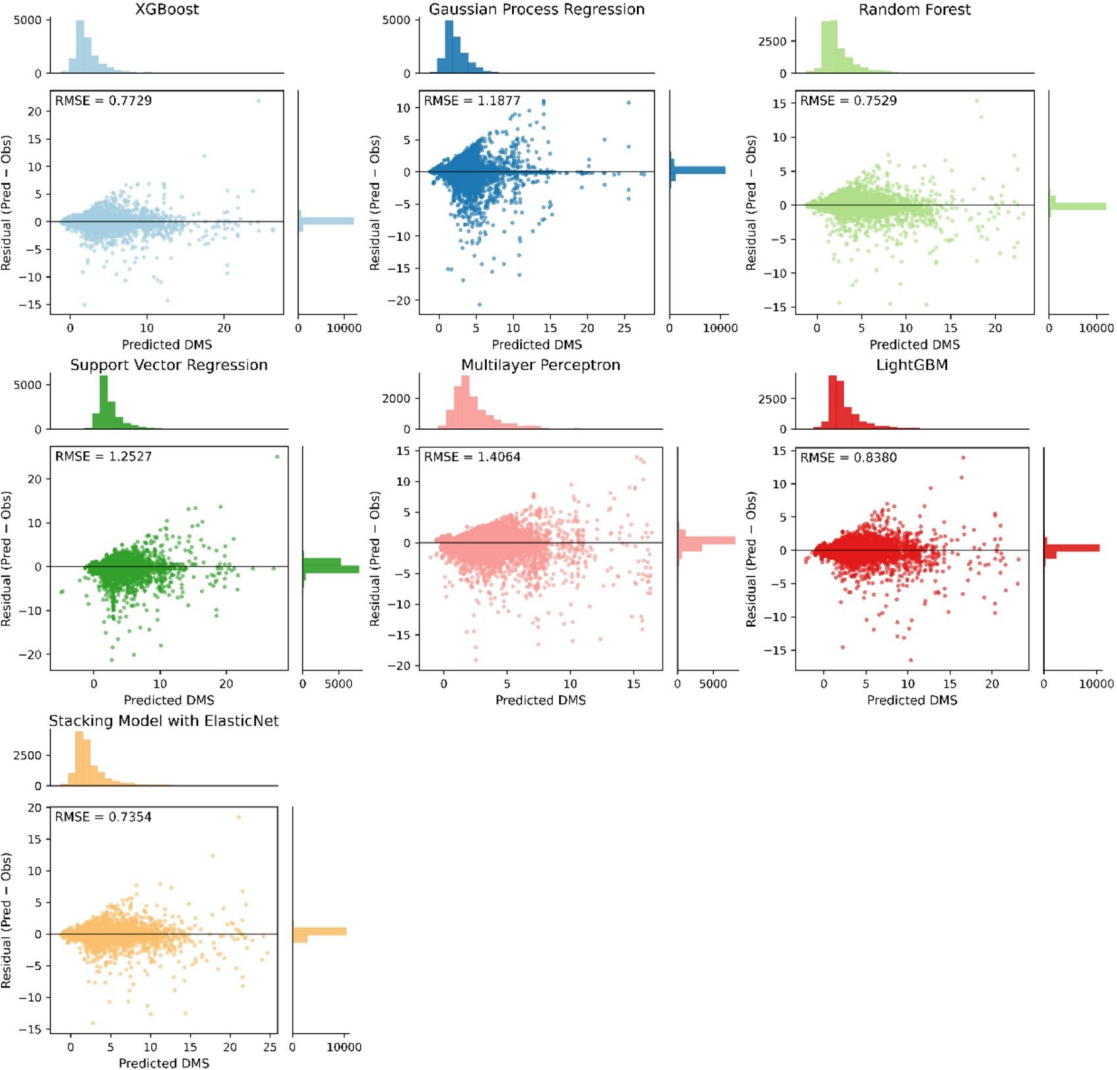
Residual distributions reveal distinct error patterns across models. Residuals (*Predicted - Observed~DMS*) plotted against predicted DMS concentrations. The stacking ensemble exhibits the smallest RMSE (0.721 µmol m^−3^) and the most stable residual spread. Tree-based models (XGBoost, Random Forest, LightGBM) exhibit moderate scatter without clear patterns, while kernel-based methods (GPR, SVR) and neural networks (MLP) show substantial heteroscedasticity with increasing errors at higher DMS concentrations.

**Fig. 5:**
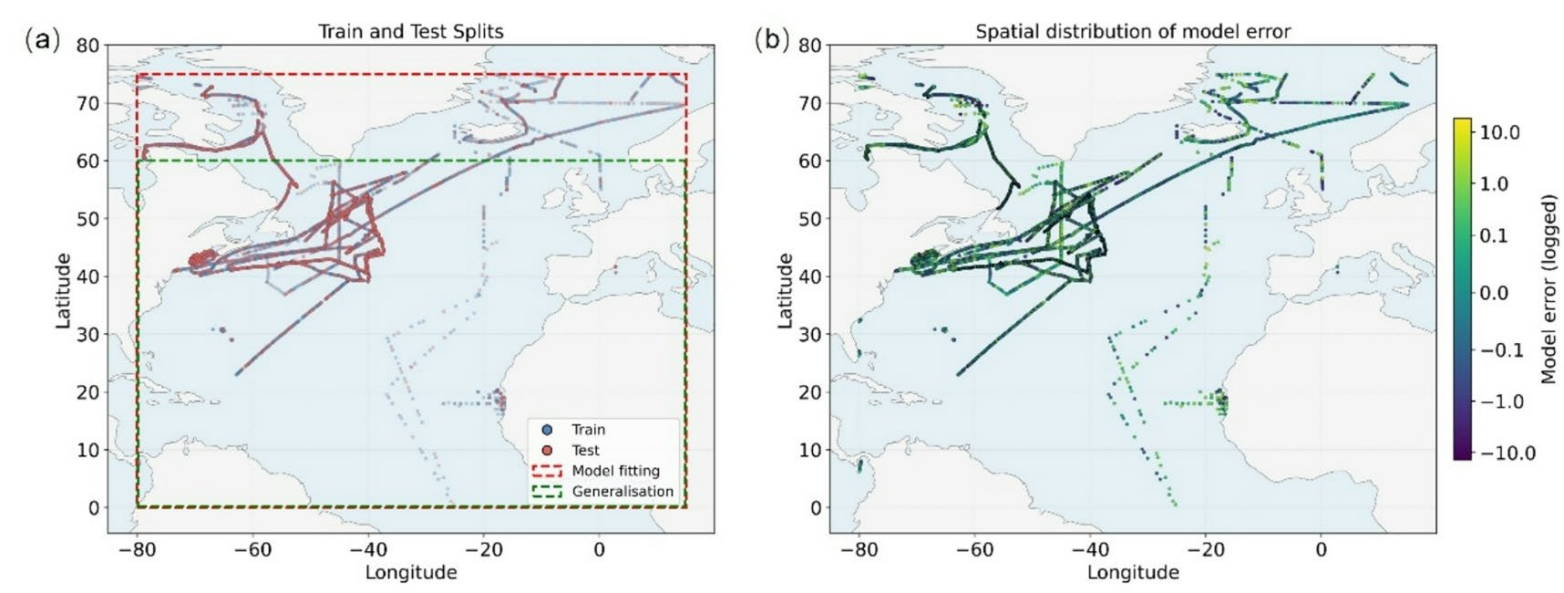
Spatial distribution of the training and test subsets and the corresponding test-set absolute errors. **a** Spatial distribution of in situ DMS observations in the training and test subsets. **b** Spatial distribution of absolute prediction error of the stacking ensemble. Maps were produced by the authors using Python [3.10.18], Cartopy [0.23.0]^71^ and Matplotlib [3.5.3]^72^. The basemap features were obtained from Natural Earth (License: Public Domain)^73^ via Cartopy.

Residual analysis further confirmed these patterns (Fig. 4). The ElasticNet stacking model exhibited the narrowest residual distribution and minimal heteroscedasticity, whereas kernel-based and neural network models showed increasing error variance with predicted concentration. The residuals of all models are normally distributed with a mean of zero (Supplementary Fig. S10). To further assess the representativeness of the dataset split, we mapped the spatial distributions of the training and test sets (Fig. 3a). Despite the sparse distribution of ground-truth observations, the random split produced comparable spatial coverage between the two subsets. The temporal split was also evenly distributed (Fig. S21). We also mapped the absolute prediction error (Fig. 5b), which showed no strong spatial clustering, suggesting that model performance was not driven by a few specific regions and that the chosen split was not associated with obvious spatial bias.

As an interpretable proxy for model’s predictive uncertainty, we quantified the standard deviation (SD) of predictions across the six base learners. This inter-model spread showed a moderate positive correlation with the absolute error of the ensemble prediction (Pearson *r* = 0.558; Spearman *ρ* = 0.523). The spread distributed with a median of 0.23 and an interquartile range of 0.14–0.45, although a small number of samples showed substantially larger disagreement among models (Fig. S17). When samples were grouped into quartiles according to this spread, the mean absolute error of the stacked model increased monotonically from 0.107 in the lowest-spread quartile to 0.768 in the highest-spread quartile. This means that the greater the variation among the base learners, the greater the error in the ensemble. Disagreement among base models provides a useful and interpretable indication of uncertainty in the final estimation.

### Daily DMS dataset for the North Atlantic (2002–2024)

#### Dataset characteristics

Using the validated ElasticNet stacking model, we generated a daily DMS dataset for the North Atlantic for over 20 years (spanning from 2002 to 2024). The dataset provides DMS estimates at 4 km spatial resolution over 0–60°N and 80°W-15°E, covering oceanographic regimes from subtropical gyres to subpolar frontal systems. Spatial coverage is constrained at higher latitudes by limitations in satellite ocean colour retrieval during polar night conditions associated with high solar zenith angle, low solar elevation, and sea ice.

The dataset comprises approximately 4.4 million grid cells per day over 8,025 days, producing 35.34 billion individual DMS estimates. Daily gridded maps are provided for all time steps.

**Fig. 6:**
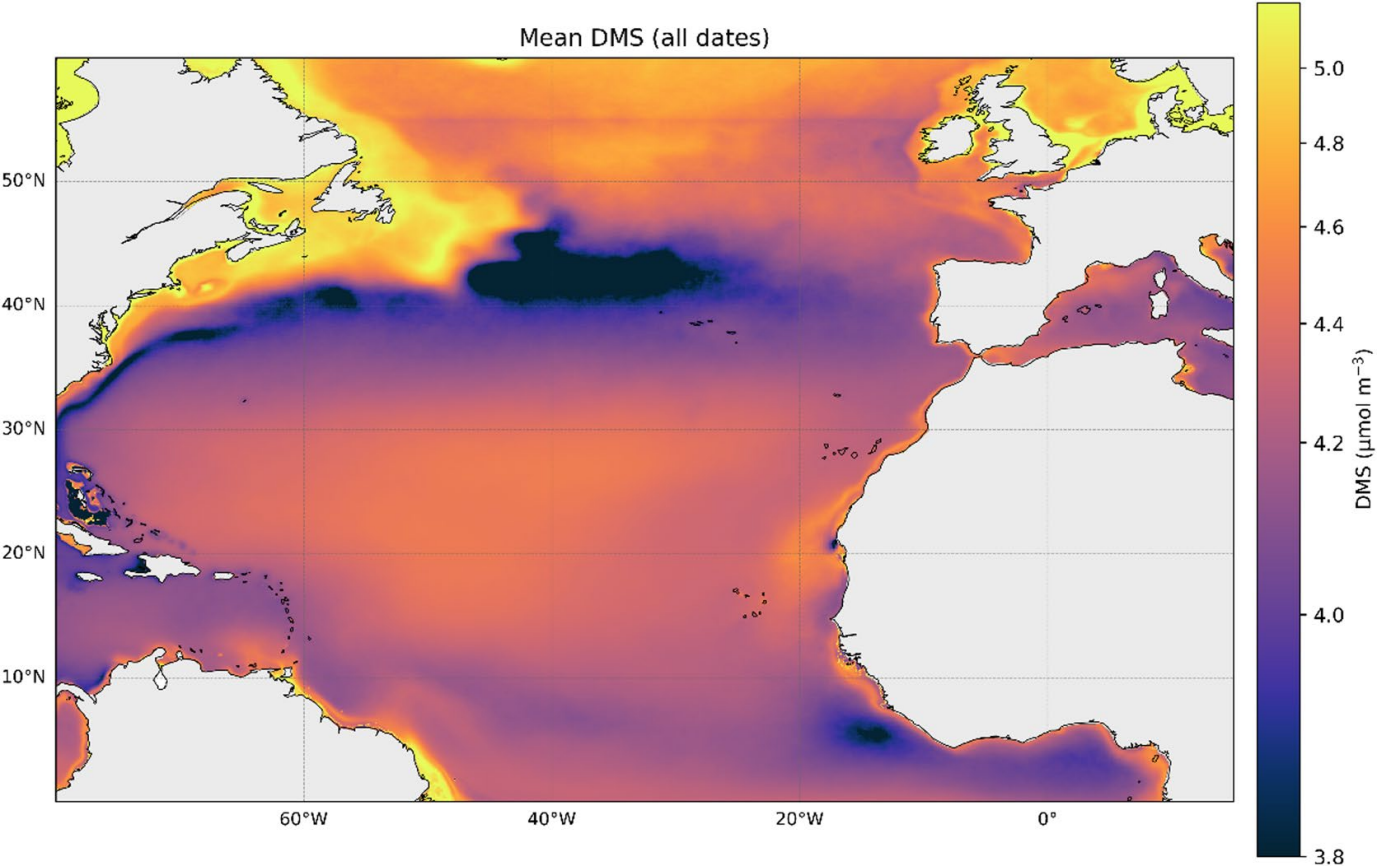
Mean spatial distribution of DMS across the North Atlantic (2002–2024). Map of time-averaged DMS concentrations based on 8,025 daily fields. Higher concentrations are observed in productive coastal and shelf regions, while subtropical and Mediterranean regions exhibit lower values. Maps were produced by the authors using Python [3.10.18], Cartopy [0.23.0]^71^ and Matplotlib [3.5.3]^72^. The basemap features were obtained from Natural Earth (License: Public Domain)^73^ via Cartopy.

Basin-wide mean DMS concentrations exhibit pronounced spatial heterogeneity (Fig. 6). Elevated values (> 4.8 µmol m^−3^) occur consistently in coastal upwelling zones, shelf regions around the British Isles and North Sea, and major frontal systems, whereas subtropical gyres and the Mediterranean Sea show persistently lower concentrations. The 4 km resolution resolves mesoscale features such as shelf-break fronts and eddies that are not captured in coarser climatological products.

#### Spatio-temporal dynamics and seasonal variability

**Fig. 7:**
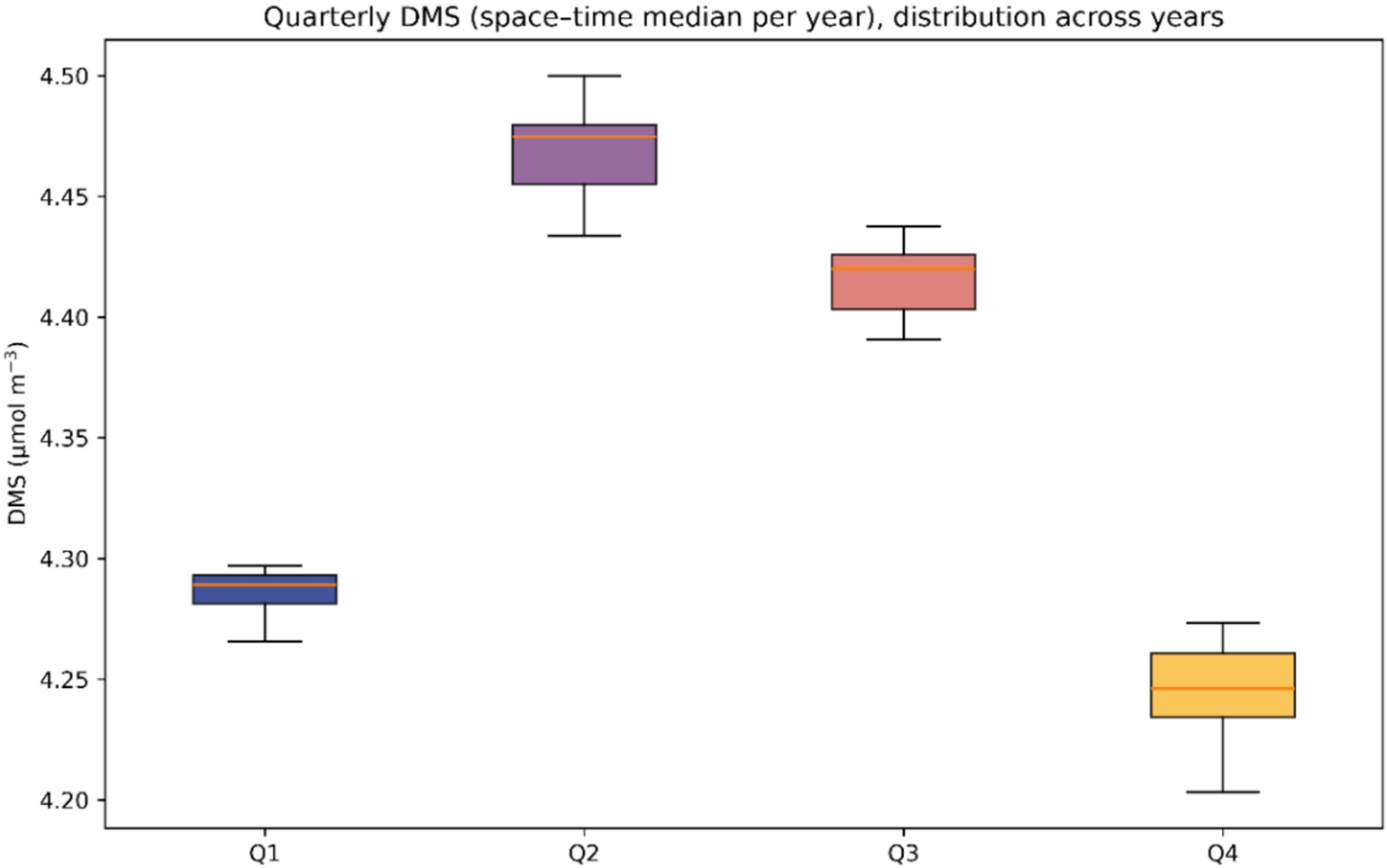
Seasonal variation in basin-averaged DMS concentrations. Quarterly distributions of spatially averaged DMS across all years. Peak values occur in Q2, with minima in Q4. Monthly trends are shown in Supplementary Fig. S11.

The dataset captures a coherent seasonal cycle consistent with North Atlantic phytoplankton phenology (Fig. 7). Spatially averaged DMS concentrations peak in late spring and early summer (Q2 median = 4.47 µmol m^−3^, IQR = 4.45–4.48 µmol m^−3^), decline through summer and autumn (Q3 median = 4.41 µmol m^−3^), and reach annual minima in winter (Q4 median = 4.24 µmol m^−3^). Early spring shows intermediate concentrations (Q1 median = 4.29 µmol m^−3^). The seasonal amplitude between Q2 and Q4 is 0.23 µmol m^−3^, corresponding to approximately 5% of the annual mean.

**Fig. 8:**
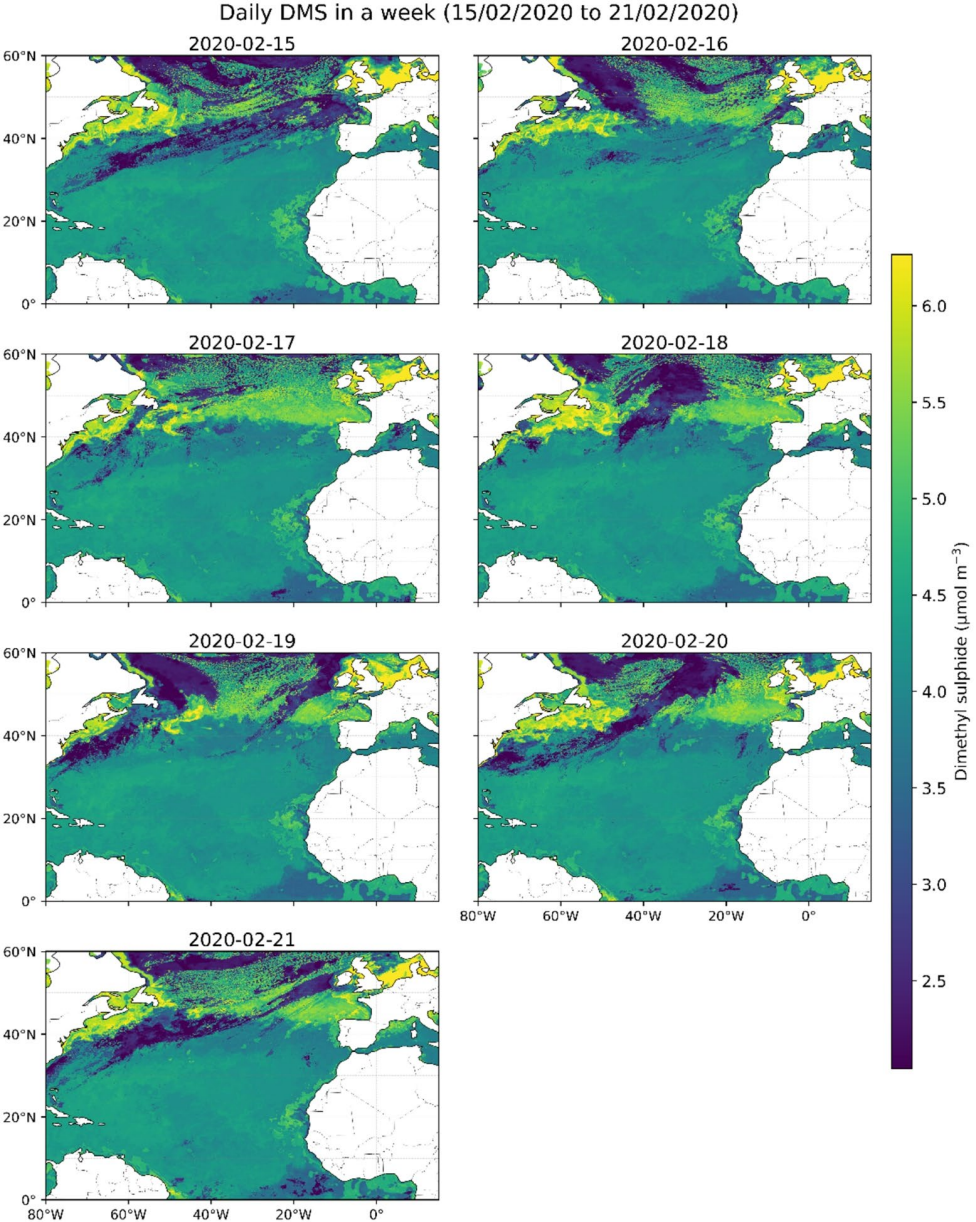
Daily Dimethyl Sulphide (DMS) concentrations across the North Atlantic for seven consecutive days (15–21 February 2020), illustrating the high temporal resolution and dynamics of the dataset. Day-to-day variability is evident, particularly along the Gulf Stream boundary and in the mid-to-high latitudes, where concentrations fluctuate between ~ 2.5–3.0 µmol m^−3^ and regional maxima exceeding 6 µmol m^−3^ within the same week. Maps were produced by the authors using Python [3.10.18], Cartopy [0.23.0]^71^ and Matplotlib [3.5.3]^72^. The basemap features were obtained from Natural Earth (License: Public Domain)^73^ via Cartopy.

A key advantage of the present dataset over existing data products is its daily temporal resolution, which enables the characterisation of short-term DMS variability that is otherwise obscured by temporal averaging. Figure 8 illustrates seven consecutive days of DMS concentration across the North Atlantic in February 2020, revealing significant day-to-day fluctuations within a single week. Along the Gulf Stream boundary and in mid-to-high latitudes, concentrations shift markedly between days, with regional maxima exceeding 6 µmol m^−3^. These dynamics, likely driven by episodic upwelling or short-lived phytoplankton responses to physical forcing, would be entirely smoothed out in monthly or seasonal composites. The dataset, therefore, captures a dimension of DMS variability that long-term climatologies from alternative sources cannot resolve.

At longer timescales, seasonal spatial patterns reveal marked regional structure (Supplementary Fig. S12). Northern temperate and subpolar regions (> 50°N) exhibit strong spring enhancement, with Q2 median concentrations frequently exceeding 5 µmol m^−3^ during bloom periods. These patterns are consistent across representative early, mid, and recent years, indicating robust seasonal dynamics despite inter-annual variability. In contrast, subtropical regions display muted seasonality, maintaining relatively stable concentrations between 3.5 and 4.5 µmol m^−3^ year-round. Coastal regions show persistent localised DMS hotspots, including the North Sea, waters surrounding the British Isles, and the Labrador Shelf, which remain spatially coherent across seasons and years.

Inter-annual variability was moderate compared to seasonal cycles, with basin-mean DMS concentrations ranging from 4.316 µmol m^−3^ (2018) to 4.372 µmol m^−3^ (2012), representing a narrow range. Visual comparison of spatial patterns across years (Supplementary Fig. S12-S13) reveals that while basin-averaged values varied modestly, regional anomalies were more remarkable. For instance, 2023 Q1 showed enhanced DMS in the Labrador Sea relative to 2003 and 2015, while the subtropical North Atlantic remained consistently stable across all years. Linear regression revealed no significant long-term trend over the 22-year period (slope = 0.0001 µmol m^−3^ yr^−1^, R^2^ = 0.001, Fig. S18), indicating relatively stable basin-averaged DMS levels despite documented changes in North Atlantic oceanographic conditions such as warming and circulation shifts. Nevertheless, individual years and quarters exhibit notable anomalies, including elevated DMS in 2012 and suppressed concentrations in 2018.

#### Comparison with existing DMS models

We compared the ElasticNet stacking model against existing machine learning and empirical DMS products developed for regional and global applications. Our model demonstrated substantial improvements over existing approaches across multiple metrics.

Among machine learning methods, Mansour et al.^47^ developed a Gaussian Process Regression (GPR) model for the North Atlantic (1998–2021) at 0.25° spatial resolution, achieving R² = 0.71. Wang et al.^44^ created a global monthly DMS climatology by an artificial neural network (ANN), capturing 66% of observed variance (R² = 0.66). In the northeast subarctic Pacific, McNabb and Tortell^46^ achieved R² = 0.62 using ensemble random forest and ANN models.

Our ElasticNet stacking ensemble substantially outperforms these regional and global ML approaches, achieving test R² = 0.88 and overall R² = 0.92 on all observation points, representing a significant improvement over the best previous North Atlantic model and global models.

Empirical and interpolation-based products exhibit larger discrepancies. Galí et al.^38^ satellite-based algorithm (G18) achieved R² = 0.50. Lana et al.^42^ interpolation-based climatology (L11), derived from binned in-situ measurements, remains a benchmark but captures limited temporal variability due to its monthly resolution and spatial averaging. Widely used climatologies typically operate at monthly resolution and exhibit RMSE values exceeding 1.0 µmol m^−3^. Our model achieves a test RMSE of 0.859 µmol m^−3^ and an overall RMSE of 0.721 µmol m^−3^, indicating markedly improved precision.

In addition to accuracy, the principal advancement of our model lies its spatiotemporal resolution. The 4 km daily product represents a sevenfold spatial refinement relative to the best previous North Atlantic machine-learning model and a more than an order-of-magnitude improvement over global monthly climatologies. This resolution enables explicit representation of mesoscale fronts, coastal hotspots, and short-lived bloom events that are smoothed or absent in coarser products. These fine-scale features are likely critical for ecological applications, particularly for seabirds that encounter DMS gradients over kilometre to tens-of-kilometre scales during daily movements.

Moreover, the daily temporal resolution enables capture of algal bloom events and sub-weekly DMS dynamics that are critical to understanding olfactory navigation cues but are smoothed out in monthly climatologies. Spatial pattern comparisons with Mansour et al.^47^ and G18^38^ reveal broad agreement on large-scale features: High DMS in subpolar regions (> 50°N) during spring-summer, suppressed concentrations in subtropical gyres, but notable divergence in fine-scale coastal features and frontal zones. Also, our results and the modelling results from these studies agree with the earliest modelled DMS distribution in Kettle et al.^48^. Our 4 km resolution resolves persistent coastal hotspots and mesoscale frontal structures that appear as diffuse gradients in coarser products. These fine-scale features, invisible in coarse climatologies, likely represent ecologically significant DMS signals accessible to foraging seabirds.

### AniDMS for trajectory-DMS integration

#### Package functionality and workflow

**Fig. 9:**
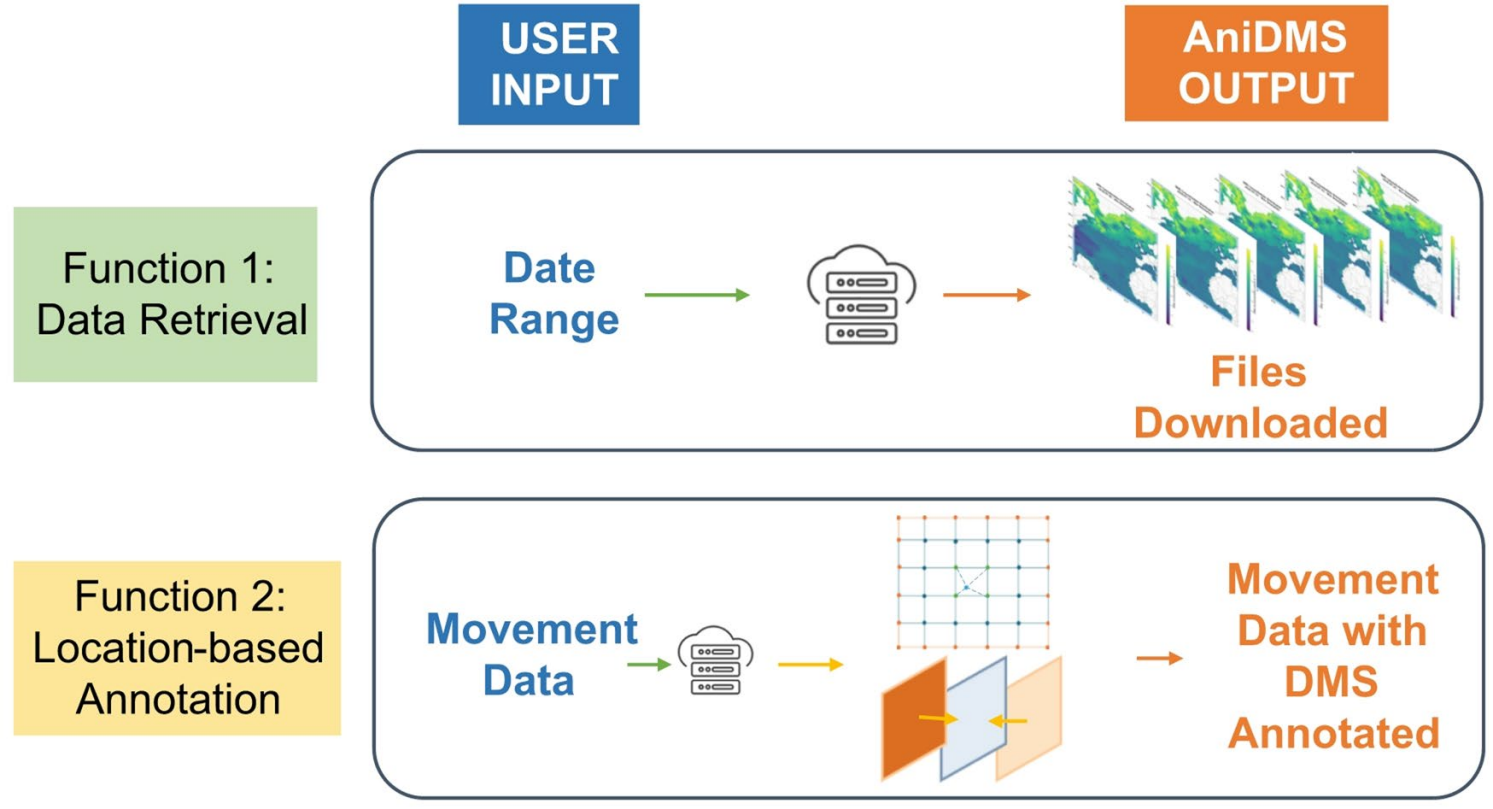
Workflow of the AniDMS tool. AniDMS provides database query functionality for retrieving daily DMS fields and automated trajectory annotation through spatial-temporal interpolation.

The daily DMS data products generated in this study are archived and publicly available via Zenodo^49^. To facilitate efficient access and integration into ecological workflows, we developed AniDMS, an open-source Python package that implements a lightweight tool for providing access to the DMS dataset and automated annotation of movement trajectories (Fig. 9).

AniDMS offers two core functions. First, a database query function allows users to specify a date range and automatically retrieve the corresponding daily DMS fields for the entire North Atlantic from Zenodo, avoiding manual navigation or bulk downloads of the full archive. Second, a trajectory annotation function enables extraction of DMS values along movement paths. Users provide trajectory data containing timestamps and geographic coordinates, and AniDMS matches each point to the corresponding daily DMS data, extracting values via inverse distance weighting interpolation and linear temporal interpolation as described in Sect. 5.2. As daily timestamp is set at 12 pm in DMS data, spatial interpolation is done for both of the previous and later days, and a linear temporal interpolation based on the time of trajectory points is carried out to obtain the annotated DMS value.

For spatial interpolation, geographic coordinates are internally transformed to the Lambert Azimuthal Equal-Area projection, and DMS values are computed from the nearest grid cells using adaptive neighbourhood expansion to ensure robust extraction in regions with sparse valid pixels. Annotated DMS values are appended directly to the input trajectory dataset, and the coordinates are transformed back to geographic coordinates before it returns to the user. The package is lightweight and platform-independent, which can be installed either via Conda or Python Package Index, requiring only an internet connection and standard Python dependencies.

#### Case study: DMS annotation of Manx shearwater trajectories

**Fig. 10:**
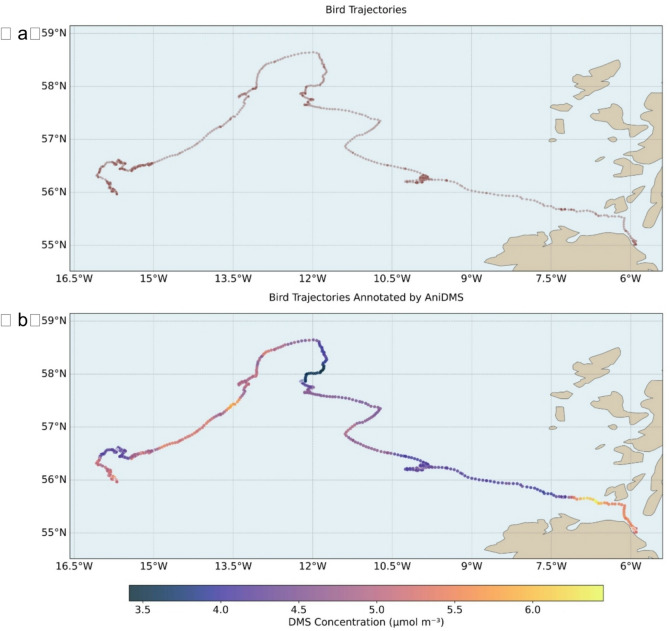
Application of AniDMS to Manx shearwater trajectories. **a** Representative GPS tracking data from breeding Manx shearwaters on Copeland Island. **b** July 2019 trajectories annotated with daily DMS concentrations. Colours indicate interpolated DMS values (µmol m^−3^) along individual movement paths. Maps were produced by the authors using Python [3.10.18], Cartopy [0.23.0]^71^ and Matplotlib [3.5.3]^72^. The basemap features were obtained from Natural Earth (License: Public Domain)^73^ via Cartopy. Only the colours of the basemap features were customised.

Using AniDMS, daily DMS concentrations were extracted for each trajectory point. Annotated trajectories from July 2019 reveal pronounced spatial heterogeneity in DMS exposure (Fig. 10b). Birds foraging in offshore pelagic waters experienced relatively uniform moderate concentrations (approximately 3.5–4.5 µmol m^−3^), whereas individuals operating in coastal and shelf regions encountered elevated and highly variable DMS fields. Fine-scale gradients were clearly resolved, with individual birds traversing concentration changes of 1–2 µmol m^−3^ over distances of 10–20 km.

The trajectory for demonstration was subset from July 2019, comprising 1530 trajectory points for 2 individuals, was processed in under one minute on a standard laptop, illustrating the efficiency of the annotation workflow. The 4 km daily resolution of the underlying DMS product enabled alignment of environmental conditions with movement decisions at biologically relevant scales. Comparable patterns would be obscured in monthly or coarse-resolution climatologies. This case study, therefore, establishes AniDMS as a practical tool for integrating high-resolution DMS fields into movement ecology analyses. Detailed investigation of relationships between DMS exposure patterns and foraging behaviour, navigation strategies, and breeding success will be carried out in subsequent studies.

## Discussion

This study provides the first spatially continuous, high resolution dimethyl sulphide dataset for the North Atlantic Ocean at daily temporal resolution and 4 km spatial resolution, covering the period 2002 to 2024. With AniDMS, a package providing tools for automated data retrieval and annotation, this work bridges a long-standing mismatch between coarse resolution DMS climatologies and the spatiotemporal scales at which marine predators experience and respond to their chemical environment. By integrating ensemble machine learning with extensive in situ observations, we enable both improved environmental reconstruction and direct ecological application.

### Model performance and methodological advances

The stacked ensemble model demonstrates strong predictive performance across the North Atlantic domain. On the test data, the model achieves an R² of 0.88 and an RMSE of 0.859 µmol m^−3^, substantially exceeding previously reported basin-scale models for DMS, which typically achieve R² values around 0.7 or lower^38,44,46,47^. The strong performance retained on the holdout test set indicates good generalisation. When applied to the full spatiotemporal prediction domain, the model attains an overall R² of 0.915 with an RMSE of 0.721 µmol m^−3^, which is only slightly lower than performance on the training set. This means that the model shows good generalisation. Also, model performance was evaluated using a random train-test split. Inspection of the resulting subsets showed that both training and test samples remained well distributed across space and time, and prediction errors did not exhibit obvious spatial pattern, suggesting that the reported accuracy is representative across the domain.

The achieved 4 km daily resolution represents a major refinement relative to existing global or regional products that are monthly and typically exceed 25 km in spatial resolution^39,42,47^. This improvement enables the resolution of transient bloom events and sharp spatial gradients that are systematically smoothed in climatological products. Such fine-scale variability is particularly relevant for mobile organisms that integrate environmental cues over hours to days rather than monthly averages.

Methodologically, the improved performance arises from ensemble stacking that leverages complementary strengths across diverse algorithms, combined with rigorous cross-validation and conservative model selection. Importantly, SHAP-based interpretation identifies mixed layer depth, nitrate, chlorophyll and temperature as dominant drivers, consistent with established mechanistic understanding of DMS production and release. This alignment between statistical attribution and biogeochemical theory provides confidence that the model captures meaningful environmental relationships rather than purely empirical correlations.

Residual diagnostics indicate minimal systematic bias across latitude bands and productivity regimes, with robust performance from subtropical oligotrophic waters to subpolar bloom-dominated regions. The ensemble framework also mitigates heterogeneity in source observations derived from multiple analytical protocols, as weaker individual learners contribute complementary nonlinear structure within the stacked model. This combination of performance and interpretability directly addresses common criticisms of machine learning approaches as opaque or ecologically disconnected.

### Ecological significance and applications

Our dataset reveals clear seasonal and spatial structure consistent with North Atlantic phytoplankton phenology. DMS concentrations peak during late and spring and decline to winter minima. Persistent coastal hotspots and strong latitudinal gradients are evident, with subpolar regions frequently exceeding 5 µmol m^−3^, while subtropical gyres remain below approximately 4.2 µmol m^−3^ throughout the year. Interannual variability is modest, with no detectable long-term trend over the study period, although individual seasons exhibit anomalies plausibly linked to climate variability.

These fine-scale patterns are particularly relevant for seabird ecology. *Procellariiform* seabirds are known to rely on olfactory cues during navigation and foraging, with DMS widely hypothesised as a key signal^22–25^. Previous empirical tests of this hypothesis have been constrained by the coarse resolution of available DMS products, which are poorly aligned with the spatial and temporal scales of individual movement decisions. Monthly climatologies at tens of kilometres resolution obscure gradients that birds may encounter and respond to within single foraging trips.

The Manx shearwater case study illustrates this limitation directly. Individuals routinely traverse DMS gradients of 1 to 2 µmol m^−3^ over distances of 10 to 20 km within a single day. Such gradients are entirely smoothed in existing climatological products but are clearly resolved in the present dataset. AniDMS enables direct alignment of DMS exposure with movement trajectories, supporting rigorous tests of olfactory guided navigation, habitat selection, and foraging efficiency at biologically realistic scales.

The dataset supports broader applications, including analyses of phytoplankton bloom dynamics, air-sea sulphur flux estimation, and evaluation of biogeochemical model outputs. The modelling framework is transferable to other marine tracers or environmental variables, and the open-source design encourages reuse and extension by the wider community.

### Limitations and future directions

As with all synthesis products, the underlying observation database exhibits spatial heterogeneity, with higher sampling density in coastal and shelf regions relative to offshore areas. Surface seawater DMS measurements have been accumulated through heterogeneous ship cruises and observations rather than through a spatially and temporally uniform observing system. Earlier database studies noted both the sparsity and uneven monthly coverage of DMS measurements, which complicated the construction of gridded fields^48^. Problems such as under- or over-representation of specific seasons or regions may lead to greater uncertainty. Within the defined domain of 0–60°N, model evaluation indicates robust performance, but extrapolation beyond this range remains constrained by satellite data gaps during polar night. Temporal coverage in the ground-truth data currently spans 2003 to 2018, and long-term regime shifts outside the training distribution could introduce model drift, highlighting the need for periodic retraining if new observations become available.

An additional limitation is that not all environmental predictors are derived from satellite observations. Instead, the model estimation relies on a mixture of merged Level-4 products, reanalysed models, hindcast biogeochemical data products, and fused PAR inputs. We note that PAR estimates in persistently cloud-covered regions remain more uncertain than in directly observed areas, and the associated gap-filling procedure should therefore be interpreted as a continuity-preserving step rather than a projection of the daily field. This improves spatial and temporal completeness in the predictor set but also means that uncertainty associated with cloud-related retrieval conditions, interpolation, data assimilation, and model structure is not always represented as explicit spatial gaps, and some short-term fine-scale variability may be smoothed.

Heterogeneity in analytical methods across historical DMS measurements remains an inherent challenge for marine data synthesis. While ensemble modelling partially mitigates this issue, continued efforts toward standardisation and expanded in situ sampling will further improve reconstruction fidelity.

Future work should extend this framework to global oceans, particularly the Southern Ocean and Pacific, enabling comparative analyses of DMS-mediated navigation across regions. Integration with near-real-time satellite products could support operational forecasting, while coupling with process-based biogeochemical models would allow exploration of future climate scenarios. Ecologically, these advances would enable tests of competing navigation theories in animals, for example, by contrasting the explanatory power of magnetic, olfactory and mixed-cue strategies for return flights. High-resolution DMS data could also be used to parameterise and fit agent-based movement models that formalise alternative decision rules, thereby enabling inference across individuals and contexts. AniDMS is readily applicable to other marine taxa and environmental variables, lowering technical barriers for interdisciplinary synthesis.

## Conclusion

This study delivers the first spatially continuous dimethyl sulphide dataset for the North Atlantic at biologically relevant scales, providing daily estimates at 4 km resolution over more than two decades. Regarding our research questions, the machine learning approach effectively supports ecological exploration and interpretation at the daily 4 km resolution, supporting seabird studies and navigation research. Our data product resolves fine-scale gradients and dynamics that are absent from existing data products, and AniDMS provides an accessible route to integrate the machine-learning-derived DMS estimates with trajectory annotation, making hypothesis testing in olfactory navigation viable. This advance is achieved despite the well-known constraints of the underlying in situ DMS record, which is spatially and seasonally uneven and sparse across North Atlantic. By integrating multi-source predictors within an ensemble framework and providing a spatially continuous reconstruction, our approach helps mitigate these data limitations and delivers a practically usable DMS field for movement-scale analyses.

Using an interpretable stacked ensemble approach, the model achieves strong predictive skill. By coupling this dataset with AniDMS, enabling data retrieval and annotation in a package, we directly address a methodological bottleneck that has limited empirical tests of olfactory navigation in seabirds. The Manx shearwater case study demonstrates how high-resolution environmental reconstructions can be aligned with individual movement decisions, enabling more rigorous and mechanistic ecological inference.

This work provides a transferable framework for integrating machine-learning-based environmental reconstructions into movement ecology and marine biogeochemistry. By bridging oceanography, data science and behavioural ecology, the study advances both methodological capability and ecological understanding.

## Method

### Data sources

Based on prior North Atlantic DMS modelling studies employing multilinear regression, biogeochemical models, and machine learning approaches^35,38,45,47^, five satellite-related environmental predictors were selected: chlorophyll concentration (CHL), mixed layer depth (MLD), sea surface temperature (SST), nitrate concentration (NO_3_), and photosynthetically available radiation (PAR). These variables represent key biological, physical, and optical controls on surface DMS production and variability. To compare with recent high-performing machine-learning DMS products, we use the predictors and evaluation metrics from the Gaussian process regression (GPR) study^47^ as a reference baseline and compare our results against those of that study in subsequent analyses.

In situ DMS observations were compiled from two sources: the NOAA PMEL (Pacific Marine Environmental Laboratory) global DMS database and the NASA NAAMES (North Atlantic Aerosols and Marine Ecosystems Study) regional cruise dataset (Table 2). Combined, these datasets span from 4 July 2002 to 22 June 2024, where the time period is constrained by the temporal availability of satellite predictor variables. Despite aggregation across sources, DMS observations remain sparse and unevenly distributed, totalling 14,105 records over the 22-year period.

The predictor variables were obtained from multiple Earth observation and model-based satellite data products (Table 2). CHL and SST were derived from Level-4 analysed products, while MLD and NO_3_ were taken from reanalysis and hindcast models respectively. PAR came from daily Level-3 mapped ocean-colour products, and assembled by MODIS observations, and meteorological and ozone data, which were affected by data loss in marginal regions; therefore, they required additional gap handling. To address this, VIIRS, the successor to MODIS, was used to supplement missing or degraded MODIS data and improve continuity in daily coverage (Sect. 5.2).

Specifically, CHL was obtained from the Copernicus GlobColour product, a Level-4 merged ocean-colour product. SST was obtained from two Level-4 analysed products, CAMS SACCI Analysis L4 CDS and ESA CCI CDR, which come from the same initiative but are published by different data providers. MLD was derived from the Copernicus Global Ocean Physics Reanalysis, and NO_3_ from the Copernicus Global Ocean Biogeochemistry Hindcast based on a biogeochemical model. Because these products are distributed as analysed, reanalysis, hindcast, or Level-4 gap-free data products, they were used as provided and did not require additional gap-filling or cloud-processing within our workflow.

The North Atlantic study domain spans 80°W to 15°E. Model training, validation, and evaluation were conducted using data covering 0° to 75°N, reflecting the full latitudinal range of available in situ observations. However, the produced DMS dataset was restricted to 0° to 60°N because CHL and PAR products remain subject to persistent limitations at higher latitudes during winter, particularly under polar night, sea-ice cover, low solar elevation, and solar zenith angle constraints. Even within the retained domain, occasional wintertime CHL gaps may still occur between approximately 55° and 60°N. These limitations are inherent to high-latitude ocean-colour retrieval and are not unique to our workflow. However, this is not problematic for our ecology case, as the operational domain of 0° to 60°N corresponds to the primary activity range of Manx shearwaters and the vast majority of North Atlantic migratory seabirds. Model performance is therefore interpreted within the 0°−60°N range. These high-latitude winter gaps did not affect model training because in situ DMS observations in those conditions were limited. As a large-scale extrapolation to fill missing latitude bands is not feasible, dates and latitude bands affected by predictor-data gaps were recorded in the dataset metadata so that users can identify periods and regions where predictions are unavailable or should be interpreted with additional caution.

All predictor variables were stored as netCDF arrays^50^ on a regular geographic grid with a spatial resolution of 0.036° in the WGS84 coordinate reference system. For efficient processing, data were partitioned into spatial blocks and stored as compressed Parquet files using Dask^51^ for parallel computation.

### Preprocessing

Spatial operations involving distance calculations, including nearest neighbour searches and inverse distance weighting interpolation, were performed in projected coordinates in the Lambert Azimuthal Equal Area coordinate system. We chose this system because it preserves area and provides calculations in metres with minimal distortion across the target domain when centred at 37.5°N, 32.5°W. The whole preprocessing workflow is demonstrated in Supplementary Fig. S15. The same pipeline was applied during large-scale DMS estimation to ensure methodological consistency.

Gaps in predictor variables were addressed using strategies associated with the specific variables. Since the L4 data products are continuous and gap-free, CHL, MLD, NO_3_, and SST do not require additional gap-filling. PAR required a separate data fusion because MODIS-Aqua PAR contained substantial missing data due to sensor coverage and orbital constraints. Aqua MODIS gaps were first filled using Terra MODIS observations after applying land masks. VIIRS PAR was then used as an additional auxiliary source for the remaining missing areas because it provides a closely compatible continuation of the MODIS ocean-colour record. These L3 products from NASA were mapped uniformly originally and did not require grid alignment. This is further supported by NASA’s multi-mission processing framework and previous ocean-colour studies showing that multi-sensor merging is a standard way to improve spatial and temporal coverage while preserving consistency^52–54^. This procedure substantially reduced missing coverage prior to subsequent interpolation steps. Map comparison of gap-filling is included in Fig. S22. The remaining small spatial gaps were interpolated using inverse distance weighting. Satellite maintenance events resulted in complete data gaps lasting approximately one to two weeks in 2003 and 2022; these irrecoverable periods are documented in our DMS database metadata. For a small number of extended PAR gaps explored during pilot experiments, temporal interpolation combining wavelet transformation and Gaussian filtering was applied by first deriving daily mean or median series, depending on the data distribution, to represent the broad temporal trend, and then adapting this trend with 8-day smoothed products (Supplementary Fig. S14). This procedure was exploratory and was not part of the primary workflow used for the final dataset.

### Machine learning model development

**Fig. 11:**
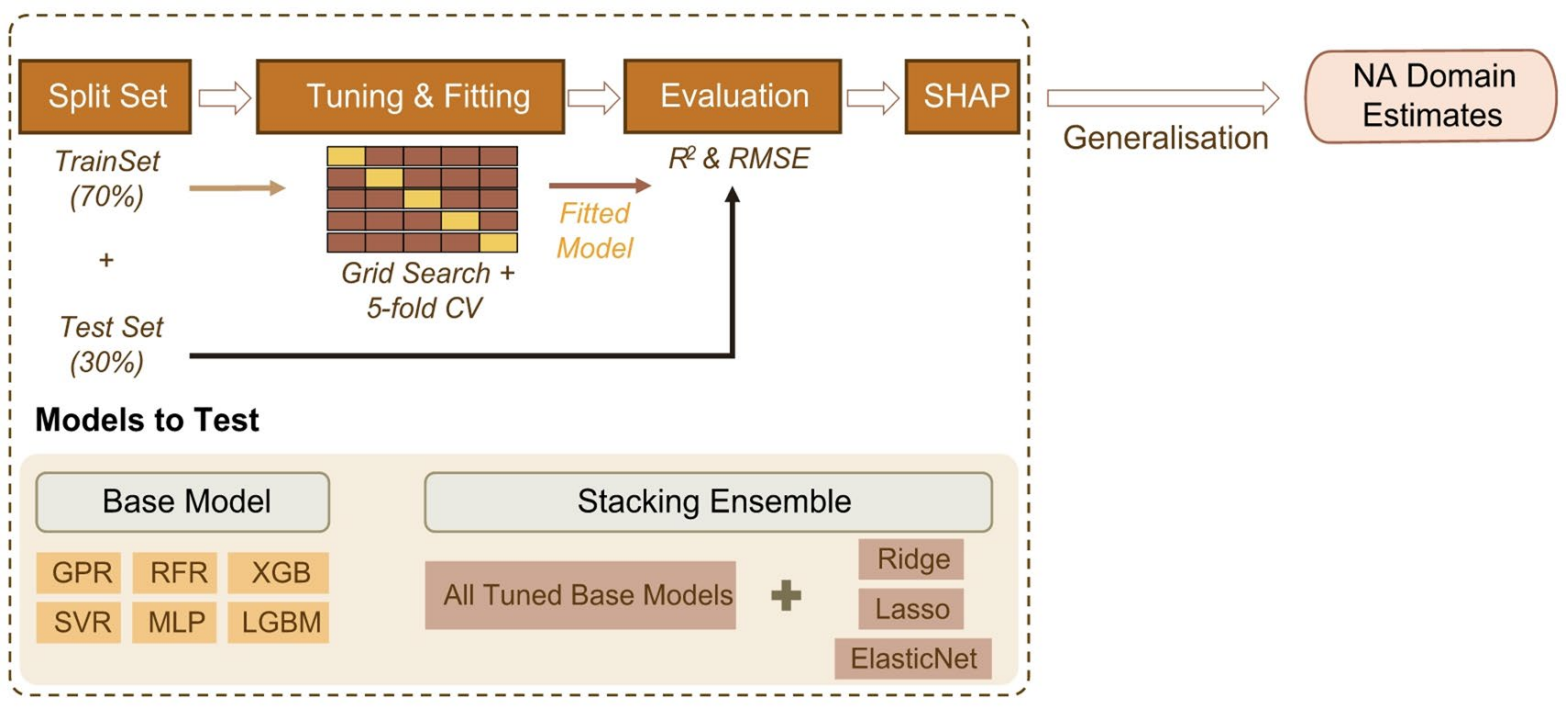
Machine Learning workflow for DMS estimation. The machine learning approach applied 6 base models and 3 ensemble models to test performance. The train set was used for model tuning and fitting, and the fitted model was evaluated on the test set. The fine tuning was composed of grid search and 5-fold cross validation. After tuned and evaluated, a SHAP analysis was conducted to evaluate feature dependence. Same as preprocessing, the pipeline for producing DMS estimates was demonstrated in Supplementary Fig. S15.

The workflow of model training and testing is included in Fig. 11. We split the dataset into training (70%) and test (30%) sets. All samples were split using a fixed random seed of 42 to ensure reproducibility. The spatial distribution of the resulting subsets is shown in Fig. 3a. Although the ground-truth DMS observations were sparse over the 22-year period, the set splits remained well-distributed spatially and temporally (Fig. S21). Time series were not included in the modelling process. The training set was used only for model development, including hyperparameter tuning and final model fitting. The test set was kept fully independent and was used only for the final evaluation of predictive performance. Given the disparate numerical scales of predictor variables, we standardised the training set using a standard scaler to ensure balanced influence during model fitting. To prevent information leakage, standardisation was based only on the training data, and the fitted scaler was subsequently applied to the validation folds and test set without refitting.

**Table 2:** Data sources used in this study. Processing levels follow remote-sensing conventions, where Level 3 (L3) denotes gridded satellite-derived products and Level 4 (L4) refers to gap-filled or model-integrated datasets.

Variable		Source product		Product type/level	Coverage and resolution
Response variable	DMS	NOAA-PMEL^55^		In-situ measurement	1972–2019, Global
		NASA-NAAMES^56^			2016–2018, NW Atlantic
Predictor variable	CHL	Copernicus GlobColour (OCEANCOLOUR_GLO_BGC_L4_MY_009_104)^57^		L4	1997–2025, Daily, 4 km
	MLD	Copernicus Global Ocean Physics Reanalysis (GLOBAL_MULTIYEAR_PHY_001_030)^58^			1993–2025, Daily, 4 km
	NO_3_	Copernicus Global Ocean Biogeochemistry Hindcast (GLOBAL_MULTIYEAR_BGC_001_029)^59^			1993–2025, Daily, 0.25°
	SST	Copernicus Atmosphere Monitoring Service	SACCI Analysis L4 CDSv2.1^60^		1979–2022, Daily, 4 km
		The European Space Agency (ESA) Climate Change Initiative	CDRv3.0^61^		1980–2024, Daily, 4 km
	PAR	NASA Ocean Colour	MODIS Aqua^62^	L3 Mapped	2002–2024, Daily with gaps, 8-day gap-free, 4 km
			MODIS Terra^63^		
		NOAA-20	VIIRS^64^		2017–2025, Daily, 4 km
Manx shearwater trajectory		Field work (GPS Employed)			Around a decade

#### Model implementation and hyperparameter optimisation

We evaluated six machine learning algorithms representing complementary modelling paradigms: XGBoost (XGB)^65^, Gaussian Process Regression (GPR)^66^, Random Forest Regression (RFR)^67^, Support Vector Regression (SVR)^68^, Multi-Layer Perceptron (MLP)^69^, and lightGBM (LGBM)^70^.

The algorithms can be categorised as tree-based ensemble methods, kernel methods and neural network. Tree-based ensemble methods (XGB, RFR, LGBM) were selected for their ability to capture nonlinear interactions and threshold effects in environmental predictors (Fig. S19). XGB and LGBM employ gradient boosting with different tree growth strategies, while RFR uses bootstrap aggregation to reduce variance. Model complexity was controlled through depth constraints and L1 and L2 regularisation.

Regarding kernel-based models, GPR was included to provide a flexible nonparametric baseline with explicit uncertainty structure, while SVR was applied using kernel-based regression with ε-insensitive loss to capture smooth nonlinear relationships in high-dimensional feature space. Lastly, MLP was implemented as a feed-forward neural network with a maximum of four hidden layers to balance model capacity and stability.

To manage computational cost, kernel selection (RBF, Matérn, Rational Quadratic) and hyperparameter tuning were conducted on a stratified subset of 6,000 observations. Hyperparameters are pre-specified parameters used to control the learning process. Model tuning involves conducting hyperparameter optimisation, which aims to find the best-performing hyperparameter set for the model. For all base models, grid search with 5-fold cross-validation on the training set was performed, with mean squared error as the optimisation criterion. Random search was evaluated during pilot testing but performed poorly. As our focus was the robustness of DMS estimation, model performance was assessed on an independent test set using mean squared error (MSE), root mean squared error (RMSE), and R² as metrics to compare and determine the most appropriate model.

#### Ensemble stacking framework

To exploit complementary strengths across base learners and balance overfitting and generalisability, we implemented a stacking ensemble framework (Fig. 11). The ensemble was constructed as a two-stage supervised learning procedure in which a meta-learner was trained on the base learners’ predictions.

In the first stage, the training set was divided into five folds. For each fold, each base learner was trained on the remaining four folds and used to predict the hold-out fold. Repeating this process across all five folds generated a complete set of out-of-fold predictions for each training sample from each base model. These out-of-fold predictions were then concatenated to form the meta-feature matrix for second-stage learning. These meta-features capture diverse nonlinear representations of the underlying regression relationship.

In the second stage, the meta-feature matrix, together with the original predictor variables, was used to train a regularised linear meta-learner. This design allowed the ensemble to integrate both the diverse nonlinear representations captured by the base learners and the original environmental covariates. Three regularised linear models with different regularisation were evaluated, and their details were illustrated in Supplementary Fig. S16.

Similarly, for predictions on the test set, each base learner was refitted on the full original training set, then applied to the test set to generate first-level predictions. These first-level predictions were concatenated in the same order as the training-stage and combined with the original predictors before being passed to the trained meta-learner. The test set was not used at any stage of base-learner fitting, meta-feature generation for training, tuning, or meta-learner training.

#### Model interpretability

To interpret predictor contributions and model behaviour, we applied SHAP (SHapley Additive exPlanations) analysis^61^, enabling assessment of variable importance and nonlinear dependencies beyond aggregate performance metrics.

### Case study: DMS annotation of Manx shearwater trajectories

We demonstrate the application of AniDMS using GPS tracking data from Manx shearwaters breeding on Copeland Island, Northern Ireland (54.67°N, 5.53°W). All GPS data from Manx shearwaters were collected after approval from Oxford University’s local Animal Welfare and Ethical Review Board, and under licence from the local Statutory Nature Conservation Bodies (SNCBs). Deployment procedures were also licensed by the British Trust for Ornithology under special methods endorsement S/6128 and carried out in accordance with guidance from the Special Methods Technical Panel (SMTP). All field methods were carried out in accordance with relevant guidelines and regulations. The methods are reported in accordance with the ARRIVE guidelines where applicable to this field-based GPS-tracking study. The dataset comprises high-resolution trajectories collected during the 2019 breeding season, with positional fixes recorded at intervals ranging from minutes to hours (Fig. 9a).

## Supplementary Information

Below is the link to the electronic supplementary material.

Supplementary Material 1

## Data Availability

The datasets generated during the current study are available on Zenodo: https://doi.org/10.5281/zenodo.18615736. All the code for pipelines is available in the GitHub repository: https://github.com/BEGIN-StAndrews/AniDMS.
