# Supplementary material for: Machine-learned dimethyl sulphide (DMS) for the North Atlantic (2002–2024) to support movement studies
Source: Sci Rep. 2026 Jun 2;16:25202. doi: 10.1038/s41598-026-55205-5 (PMC13470501; doi:10.1038/s41598-026-55205-5)
Supplement: Supplementary file 1 [file 41598_2026_55205_MOESM1_ESM.docx]

# Machine-learned dimethyl sulphide (DMS) for the North Atlantic (2002-2024) to support movement studies - Supplementary Material

**Figure S1. SHAP result consistency.**

**Figure S2. Ranked Feature importance for the ensembled stacking model with mean absolute SHAP values of the features.**

**Figure S3. SHAP summary for random forest.**

**Figure S4. SHAP summary for support vector regression.**

**Figure S5. SHAP summary for XGBoost.**

**Figure S6. SHAP summary for gaussian process regression.**

**Figure S7. SHAP summary for light gradient boost machine.**

**Figure S8. SHAP summary for multi-layer perceptron.**

**Figure S9. Observed versus predicted DMS concentrations across the North Atlantic.**

**Figure S10. Model residual density distribution.**

**Figure S11. Monthly DMS distribution across all years.**

(a)

(b)

(c)

**Figure S12.** **Seasonal and inter-annual spatial variability in DMS concentrations across the North Atlantic.** Quarterly median DMS distributions for 2003, 2015, and 2023. Northern regions show pronounced seasonal cycles with elevated spring concentrations, while subtropical regions remain comparatively stable.

**Figure S13. Quarterly and inter-annual variation of DMS distribution.** Years selected for demonstration in 2.2.2.

**Figure S14. Temporal interpolation for missing data in PAR.** Daily PAR data and 8-day average PAR data were integrated by wavelet transform. A Gaussian filter was applied to smooth the boundary of interpolation.

**Figure S15. Workflow pipeline for data preprocessing.** For all predictor variables, isolated missing days were handled through temporal interpolation to ensure continuity in the daily predictor fields. This step was applied conservatively and only where surrounding observations were available. To construct the machine learning dataset, predictor variables were aligned with in situ DMS observation locations after applying land masks. Predictor values were extracted using inverse distance weighting interpolation in the projected space.

**Figure S16.** **Workflow of the stacking ensemble used for DMS prediction.** Multiple base learners (XGB, GPR, SVR, RFR, MLP, LGBM) are first trained on the original feature set, and out-of-fold predictions are made to construct the second-level training matrix *Z*. A meta-learner, Ridge, Lasso, or ElasticNet regression, is then fitted on *Z*. The geometric constraint shapes illustrate the regularisation effects: Ridge regression imposes an L2 regularisation, which shrinks all coefficient magnitudes while retaining all predictors in the model. Lasso applies an L1 regularisation with a diamond-shaped constraint region, whose corners increase the likelihood that optimal solutions lie on the axes, allowing some coefficients to be set to zero and thus inducing sparsity. ElasticNet combines L1 and L2 penalties, producing a rounded-diamond constraint that encourages a balance between coefficient shrinkage and variable selection. The meta-learner fitted under these regularisation schemes generates the final ensemble prediction.

**Figure S17. Standard deviation across base-learner predictions versus the prediction error from the ensemble.**

**Figure S18. Interannual trend from DMS estimates.**

**Figure S19. Schematic overview of the tree-based models used to predict DMS.** Top: eXtreme Gradient Boosting (XGBoost) constructs an additive model by sequentially boosting decision trees to the residuals r1, r2, r3, ... from previous trees. Bottom left: Light Gradient Boosting Machine (LightGBM) grows the tree in a leaf-wise manner, iteratively expanding the leaf that produces the largest reduction in loss. Bottom right: Random Forest works by bootstrapping, bagging the trees, and obtaining the final prediction by averaging the individual tree outputs.

**Figure S20. Availability, uncertainty and flag layers of CHL data – Take one day as an example.** For the flag plot: 1 = Land, 2 = Interpolated.

**Figure S21. Temporal distribution of set split.**

**Figure S22. Map comparison of gap-filling.** First, MODIS-Terra is used to fill the gaps in MODIS-Aqua, then the remaining gaps are filled by the L3 product derived from VIIRS. The remaining small gaps are then interpolated with inverse distance weighting.
